# Platelet-activating factors are associated with cognitive deficits in depressed coronary artery disease patients: a hypothesis-generating study

**DOI:** 10.1186/1742-2094-11-119

**Published:** 2014-07-04

**Authors:** Graham Mazereeuw, Nathan Herrmann, Hongbin Xu, Daniel Figeys, Paul I Oh, Steffany AL Bennett, Krista L Lanctôt

**Affiliations:** 1 Sunnybrook Research Institute, Sunnybrook Health Sciences Centre, 2075 Bayview Avenue, Room FG08, Toronto, ON M4N 3M5, Canada; 2 Department of Pharmacology and Toxicology, University of Toronto, Toronto, ON, Canada; 3 Canadian Institutes of Health Research Training Program in Neurodegenerative Lipidomics, Ottawa, ON, Canada; 4 Department of Psychiatry, University of Toronto, Toronto, ON, Canada; 5 Ottawa Institute of Systems Biology and Neural Regeneration Laboratory, Department of Biochemistry, Microbiology and Immunology, University of Ottawa, Ottawa, ON, Canada; 6 UHN Toronto Rehabilitation Institute, Toronto, ON, Canada

**Keywords:** Biomarker, cognition, coronary artery disease, depression, executive function, inflammation, lipidomics, memory, neurodegeneration, platelet-activating factor

## Abstract

**Background:**

Patients with coronary artery disease (CAD) are at risk of accelerated cognitive decline, particularly those with major depression. Mechanisms for cognitive deficits associated with CAD, and the effects of depression, remain poorly understood. However, CAD is associated with inflammatory processes that have been linked to neurodegeneration, may contribute to cognitive decline, and are elevated in depression. Platelet-activating factors (PAFs) are emerging as key lipid mediators that may be central to those processes and highly relevant to cognitive decline in CAD.

**Methods:**

This cross-sectional study investigated relationships between various PAFs and cognitive performance in 24 patients with CAD (age, 60.3 ± 9.4; 70.8% male). Analyses were repeated in a subgroup of 15 patients with CAD with major depression (DSM-IV). Cognitive performance was assessed using a standardized battery and summary *z* scores were calculated based on age, sex, and education norms. Global cognitive performance was the average of domain-specific *z* scores. Plasma PAF analyses were performed using electrospray ionization mass spectrometry (precursor ion scan).

**Results:**

A greater abundance of PAF PC(*O-*18:0/2:0) was associated with poorer global cognitive performance in patients with CAD (*r* = -0.45, *P* = 0.03). In the major depressed subgroup, PAF PC(*O-*18:0/2:0) (*r* = -0.59, *P* = 0.02) as well as PC(*O*-16:0/2:0) (*r* = -0.52, *P* = 0.04), and *lyso*-PAF PC(*O*-16:0/0:0) (*r* = -0.53, *P* = 0.04) were associated with poorer global cognitive performance. A greater abundance of PAF PC(O-19:5/2:0) was associated with better global cognitive performance (*r* = 0.55, *P* = 0.03), suggesting a possible compensatory species.

**Conclusions:**

This study suggests that certain PAFs might be associated with global cognitive performance in patients with CAD, with stronger relationships observed in those with major depression. Confirmation of these preliminary findings is warranted.

## Background

Cognitive deficits are prevalent in patients with coronary artery disease (CAD) [1-4] and, accordingly, patients with CAD are at risk of accelerative cognitive decline and transition to dementia [5]. Major depression significantly amplifies the rate of cognitive decline in those with CAD [6,7], as well as worsening functional outcomes and increasing the risk of mortality [8,9]. The prevention of cognitive decline in patients with CAD may rely not only on adequate treatment of depressive symptoms, but also on a targeted inhibition of the mechanisms leading to cognitive decline in those with depression. Unfortunately, response rates to antidepressant therapies are modest in patients with CAD [10,11] and there are no consistently effective treatments to prevent cognitive decline [12]. Furthermore, the mechanisms of cognitive decline in patients with CAD, and the contributions of depression, are poorly understood.

Recent reviews have implicated aberrant lipid metabolism, inflammatory activity, and oxidative stress as pathophysiological processes involved in cognitive decline [13,14] and these same mechanisms have been associated with depression in CAD [15,16]. Persistent activation of these pathways appears to precede neurodegeneration and may contribute to cognitive vulnerability in those with depression [17]. Platelet-activating factors (PAFs) are a diverse family of potent alkylacylglycerophosphocholine (lipid) second messengers. Emerging evidence suggests that PAF activation is a hallmark of pathological disruption to membrane-based lipid signaling cascades regulating inflammatory and apoptotic activity, with various PAFs demonstrating selective pro- and anti-inflammatory and neurodegenerative effects [13,18]. Accordingly, PAF activation has been associated with the presence of CAD and with inflammation, oxidative stress, and neurodegeneration; all contemporary mechanisms for depression in CAD [19]. Furthermore, PAFs are increasingly being recognized as key participants in the neurodegenerative mechanisms associated with cognitive decline, with indications of variable effects based on species identity [13,20]. As such, PAFs may be pertinent biomarkers as well as potential mediators of cognitive decline in patients with CAD, with particular relevance to those with major depression. Despite these mechanistic links, the relationships between PAFs and cognitive performance in CAD patients with or without depression have yet to be investigated. Here, we explore a cross-sectional association between measures of cognitive performance and the plasma abundance of various PAF species for the first time using a targeted lipidomic approach in patients with CAD, with and without major depression.

## Methods

### Ethics statement

This study was approved by the research ethics boards of Sunnybrook Research Institute, University Health Network at Toronto Rehabilitation Institute, and Trillium Health Partners, and was conducted according to the principles expressed in the Declaration of Helsinki.

### Participants

Participants were approached at entry into a cardiac rehabilitation program at two local centers, the UHN Toronto Rehabilitation and Trillium Health Partners cardiac centers in Toronto, Ontario between September 2010 and May 2012. Inclusion criteria consisted of the presence of CAD (myocardial infarction, percutaneous transluminal coronary angioplasty, coronary artery bypass graft, or angiographic evidence of at least 50% stenosis in one or more major coronary arteries) with a minimum of 7 weeks post-intervention, to ensure CAD stability. Participants aged 45 to 80, who spoke and understood English, and provided written and informed consent were considered. Once included, demographic information, a detailed medical history, and concomitant medications were recorded. The use of antidepressant medication was permitted on the condition of dose stability over at least 3 months, to minimize potential confounding effects (psychological or physiological) of adjustment to these medications on the study findings. Participants were excluded if they had a history of a neurological condition, had a pre-morbid diagnosis of any Axis I psychiatric disorder other than depression, or had a significant acute medical illness (for example autoimmune condition, uncontrolled diabetes mellitus, or uncontrolled hypothyroidism).

### Schedule of assessments

Performance in specific cognitive domains was assessed using a multi-domain battery specified by the National Institute for Neurological Disorders and Stroke - Canadian Stroke Network guidelines for the study of vascular cognitive impairment [21]. This battery included tests of executive function (the Animal Naming Task, the FAS Controlled Oral Word Association Test, and the Trail Making Test Part B), memory (the California Verbal Learning Test II, immediate and delayed recall, and the Brief Visuospatial Memory Test, immediate and delayed recall), and attention and processing speed (the Digit-Symbol Substitution Test and the Trail Making Test Part A). Scores for each cognitive test were converted to a *z* score based on age, sex, education, and handedness (where applicable) norms from a representative cognitively healthy population for each test and combined for each domain into a domain composite *z* score [21,22]. Global cognition was taken as the composite *z* score of all domains.

Depressive symptom severity was measured using the 17-item Hamilton Depression Rating Scale [23] and controlled as a covariate. Patients were also examined for the presence of a major depressive episode by trained study personnel using the Structured Clinical Interview for DSM-IV Disorders - Depression scale according to the *Diagnostic and Statistical Manual of Mental Disorders (DSM) IV*[24] and those meeting the inclusion criteria were included in a subgroup analysis, of the PAF-cognition relationship with CAD patients with major depression.

### Sample collection and lipidomic analyses: lipid extraction and high-performance liquid chromatography electrospray ionization mass spectrometry

Fasting blood samples were collected at the study visit and extracted plasma was frozen at -80°C and batched for analysis. Plasma lipids were extracted using a modified Bligh and Dyer method [25] adapted, as previously described, for efficient extraction of alkylacylglycerophosphocholines (PAFs) [20,26,27]. The PAF species were analyzed using precursor ion scanning with the diagnosis fragment ion of phosphocholine head group at *m*/*z* 184, as described [20,27]. Identities were predicted for each *m*/*z* value using the online phospholipid prediction tool VaLID version 1.1 [28], and validated by analysis of mass spectrometry spectral fragmentation patterns [29] and linear regression models defining retention times of each PAF subgroup, as described previously [20].

### Statistics

The ‘peak area’ of each lipid, representing the area under the curve in counts per second, was established using Analyst version 1.4.2 (AB Sciex), normalized to the synthetic internal standard (PA_PC(13:0/0:0)_) spiked at time of extraction and multiplied by 1E6 before logarithmic transformation (base 2). The associations between PAF abundance and *z* scores from cognitive performance tests were investigated using linear regression (Pearson correlation coefficient (*r*)) (SPSS statistical software, version 13.0, Chicago, IL, USA). Significant associations between a PAF and global cognitive performance were then tested in independent covariate-adjusted models, controlling for the potential influence of age, sex, years of education, and depressive symptom severity. Participant characteristics associated with global cognitive performance in linear regression or group-comparison analyses were included in independent covariate-adjusted models as needed. As this study is intended to be hypothesis generating, the findings were not adjusted for multiple comparisons.

## Results

Ninety-five patients with CAD were assessed for study eligibility at entry to their cardiac rehabilitation program. Of those, 57 patients were excluded either because they did not agree to participate in the research (*N* = 41) or because they had begun or changed their dose of antidepressant treatment within the previous 3 months (*N* = 16). Of the remaining 38 eligible patients, 24 agreed to participate in this study (participant characteristics are summarized in Table 1). Those patients included had HAM-D scores ranging from 3 to 20, and 62.5% of the patients met DSM-IV criteria for major depression.

**Table 1:** Participant characteristics

**Characteristic**	**All CAD patients (*N*** **= 24)**	**Major depressed subgroup (*N*** **= 15)**	**Associations with Global Cognition (*N*** **= 24)**
*Sociodemographic*	Value	Value	(*t* or *r*, *P* value)
Age	60.3 (9.4)	59.6 (9.2)	-0.01, 0.98
Male (%)	70.8	73.3	-0.29, 0.78
Living alone (%)	33.3	40.0	-0.52, 0.61
White (%)	70.8	66.7	1.67, 0.20*
Years of education	14.9 (3.1)	15.3 (3.2)	0.10, 0.63
*Vascular risk factors*			
Hypertension (%)	70.8	66.7	-0.21, 0.84
Smoking history (%)	79.2	80.0	0.56, 0.58
Years smoked	27.6 (13.4)	25.3 (13.7)	-0.09, 0.70
Diabetic (%)	41.7	40.0	0.31, 0.76
Body mass index	29.8 (5.4)	28.5 (5.3)	0.11, 0.61
Dyslipidemia (%)	79.2	86.7	0.75, 0.46
Number of vascular risk factors	3.6 (1.5)	3.7 (1.5)	0.23, 0.29
VO_2_ peak (ml/(kg min))	18.5 (4.7)	19.1 (4.8)	0.17, 0.44
Systolic blood pressure (mm Hg)	121.3 (17.9)	123.9 (16.9)	-0.33, 0.11
Diastolic blood pressure (mm Hg)	74.5 (10.7)	77.3 (12.3)	-0.12, 0.57
*Cardiac history*			
Percutaneous transluminal coronary angioplasty (%)	20.8	26.7	0.74, 0.58*
Myocardial infarction or ischaemic heart disease (%)	50.0	46.7	
Coronary artery bypass graft (%)	29.2	26.7	
Median weeks since event	14.0	14.0	
First episode of CAD (%)	70.0	60.0	1.56, 0.13
*Psychometric*			
Met criteria for a major depressive episode (%)	62.5	100.0	-0.86, 0.40
History of depression (%)	45.8	53.3	0.59, 0.56
HAM-D score	12.5 (4.8)	14.9 (3.8)	-0.20, 0.35
*Concomitant medications* (%)			
Antidepressants	8.3	6.7	0.64, 0.53
Acetylsalicylic acid	75.0	80.0	1.46, 0.16
Platelet inhibitor	66.7	60.0	-1.33, 0.20
Statin	100.0	100.0	-
Anti-inflammatory	4.2	6.7	-
Anti-diabetic agents	37.5	33.3	0.45, 0.66

VO_2_, volume of oxygen; HAM-D, Hamilton rating scale for depression; *t* test and Pearson coefficient summarized associations between participant characteristics and global cognitive performance where appropriate. Data summarized using mean (with standard deviation) unless otherwise stated. *One-way analysis of variance (ANOVA) (with *F* value) performed for global cognition across ethnicity and cardiac history groups, respectively.

### Cognitive performance

Performance across all cognitive tests in this sample of patients was below comparable population norms, with mean *z* scores ranging from -0.22 to -0.55 (Table 2). Similarly, global cognition, as measured by composite *z* scores from the tested cognitive domains, was also below norms, with a mean *z* score of -0.38 (±0.62). Cognitive performance was poorer in the major depressed subgroup (Table 2).

**Table 2:** **Performance (*z *** **scores) on cognitive tasks of the NINDS-CSN cognitive battery**

**Cognitive task**	**All CAD patients (*N*** **= 24) *z *** **score (standard deviation)**	**Major depressed subgroup (*N*** **= 15) *z *** **score (standard deviation)**
**Executive function**	**-0.35 (0.70)**	**-0.36 (0.76)**
Animal Naming Task	-0.28 (0.96)	-0.31 (0.96)
FAS/COWAT	-0.25 (0.92)	-0.20 (1.06)
Trail Making Test Part B	-0.53 (0.86)	-0.58 (0.93)
**Attention and processing speed**	**-0.55 (0.78)**	**-0.64 (0.82)**
Trail Making Test Part A	-0.67 (1.00)	-0.81 (1.01)
Digit-Symbol Substitution Test	-0.43 (0.76)	-0.49 (0.80)
**Memory**	**-0.22 (0.95)**	**-0.37 (1.00)**
CVLT, immediate recall (Trials 1 to 5) (verbal)	-0.51 (1.06)	-0.76 (1.01)
CVLT, delayed recall (verbal)	-0.17 (0.93)	-0.30 (0.96)
BVMT, immediate recall (Trials 1 to 3) (visuospatial)	-0.25 (1.39)	-0.35 (1.45)
BVMT, delayed recall (visuospatial)	-0.02 (1.17)	-0.01 (1.19)
**Global cognition *z *** **score (composite of all sub-domains)**	**-0.38 (0.62)**	**-0.46 (0.59)**

BVMT, Brief Visuospatial Memory Test; CVLT, California Verbal Learning Test II; FAS/COWAT, FAS Controlled Word Association Test; NINDS-CSN, National Institute of Neurological Disorders and Stroke-Canadian Stroke Network.

### Platelet-activating factors and cognitive performance

Seventy-two phosphocholine second messengers with *m*/*z* between 450 and 600 were detected in patient plasma samples. Of these, 23 species were identified as PAFs. A significant association between a greater plasma abundance of the PAF PC(*O*-18:0/2:0) and poorer global cognitive function (composite *z* score) was observed in the total sample of patients (Table 3). That association appeared to be driven by a significant relationship between a greater plasma abundance of PC(*O*-18:0/2:0) and poorer executive function (*r* = -0.51, *P* = 0.01). Trends between a greater plasma abundance of the PAFs PC(*O*-16:0/2:0), PC(*O*-18:1/2:0), and PC(*O*-18:6/2:0) and poorer global cognitive performance were detected (Table 3).

**Table 3:** **Pearson correlations between PAFs and global cognitive performance (composite *z *** **score) in patients with CAD**

**PAF species ( *m */*z *** **peak and identities)**	**All patients (*N*** **= 24)**		**Major depressed subgroup (*N*** **= 15)**	
	**Pearson coefficient ( *r *)**	***P***	**Pearson coefficient ( *r *)**	***P***
(468.3a) PC(*O-*12:0/2:0)	-0.09	0.67	0.33	0.23
(482.4a) PC(*O-*13:0/2:0)	0.17	0.44	0.19	0.49
(496.3a) PC(*O*-14:0/2:0)	-0.22	0.31	-0.39	0.16
(494.3a) PC(*O*-14:1/2:0)	-0.22	0.30	0.26	0.36
(510.4d) PC(*O*-15:0/2:0)	0.20	0.36	-0.12	0.66
(482.4c) PC(*O*-16:0/0:0)	-0.27	0.21	-0.53	0.04*
(524.4a) PC(*O*-16:0/2:0)	-0.35	0.09*	-0.52	0.04*
(522.4a) PC(*O-*16:1/2:0)	0.06	0.78	-0.03	0.92
(520.3a) PC(*O-*16:2/2:0)	-0.08	0.72	-0.03	0.92
(532.3a) PC(*O-*17:3/2:0)	0.00	0.99	0.12	0.68
(510.4d) PC(*O-*18:0/0:0)	-0.08	0.73	-0.04	0.89
(552.4e) PC(*O*-18:0/2:0)	-0.45	0.03*	-0.59	0.02*
(508.6b) PC(*O*-18:1/0:0)	0.22	0.31	0.45	0.09*
(550.4a) PC(*O*-18:1/2:0)	-0.37	0.08*	-0.47	0.08*
(548.4b) PC(*O-*18:2/2:0)	-0.10	0.64	0.14	0.61
(546.3a) PC(*O*-18:3/2:0)	-0.15	0.50	0.19	0.50
(544.4a) PC(*O-*18:4/2:0)	0.14	0.51	0.16	0.55
(542.3b) PC(*O-*18:5/2:0)	-0.08	0.69	0.28	0.31
(540.4a) PC(*O-*18:6/2:0)	-0.40	0.05*	-0.49	0.06*
(556.4d) PC(*O-*19:5/2:0)	0.12	0.59	0.55	0.03^†^
(572.3b) PC(*O-*20:4/2:0)	0.09	0.68	0.32	0.25
(570.3e) PC(*O-*20:5/2:0)	-0.29	0.16	0.03	0.92
(568.4a) PC(*O*-20:6/2:0)	-0.16	0.46	0.14	0.61

Nomenclature adheres to the recent reorganization of ontologies for lipid classes and the curated list of lipid species identified by the Canadian Institutes of Health Research Training Program in Neurodegenerative Lipidomics (CTPNL) teams [28,30]. PC(O-20:4/2:0), for example, defines a lipid species with a phosphocholine polar head group (PC), an ether linkage at the *sn-*1 position (*O*-) with a chain of 20 carbons and 2 carbons linked by an ester linkage at the *sn*-2 position, of which the *sn*-1 chain has four degrees of unsaturation (indicated as: 4) whereas the *sn*-2 chain is characterized by fully saturated acetyl group (referred to by: 0). The number preceding species name in brackets refers to *m*/*z* of the [M + H]^+^ ion. Letters distinguish species isobaric with other lipid second messengers in the curated CTPNL phospholipid list. *Significant negative associations (*P* ≤ 0.05); ^†^significant positive associations (*P* ≤ 0.05); CAD, coronary artery disease; PAF, platelet-activating factor.

The PAF PC(*O*-18:0/2:0) remained significantly associated with poorer global cognitive performance after adjusting for age (*β*_PAF_ = -0.45, *P* = 0.03), sex (*β*_PAF_ = -0.45, *P* = 0.03), years of education (*β*_PAF_ = -0.46, *P* = 0.03), and depressive symptom severity (*β*_PAF_ = -0.46, *P* = 0.03) in independent models. None of the participant characteristics was significantly associated with global cognitive performance (Table 1), thus no characteristics were included as covariates in additional models.

### Subgroup analysis: patients with CAD with major depression

Significant associations between a greater plasma abundance of PAFs PC(*O*-16:0/2:0) and PC(*O*-18:0/2:0) with poorer global cognitive performance were observed in a subgroup of patients with CAD with major depression (Table 3). Associations with these PAFs and cognition appeared to be driven mainly by relationships with poorer executive function. The *lyso*-PAF PC(*O*-16:0/0:0) also demonstrated a significant negative association with global cognitive performance in this subgroup (Table 3). Trends between a greater plasma abundance of PAFs PC(*O*-18:1/2:0), PC(*O*-18:6/2:0), and *lyso*-PAF PC(*O*-18:1/0:0) and poorer global cognitive performance were detected (Table 3). Conversely, a greater plasma abundance of the PAF PC(*O*-19:5/2:0) was associated with better global cognitive performance in patients with CAD with depression (Table 3). Sample size precluded covariate-adjusted models in this subgroup. Neither PC(*O*-16:0/0:0) (*r* = 0.17, *P* = 0.66), PC(*O*-16:0/2:0) (*r* = -0.08, *P* = 0.83), nor PC(*O*-18:0/2:0) (*r* = -0.17, *P* = 0.66) were associated with global cognitive performance in patients with CAD without depression (*N* = 9). The PAF PC(*O*-19:5/2:0) (*r* = -0.73, 0.04) was significantly associated with poorer global cognitive performance in patients with CAD without depression.

## Discussion

These preliminary findings support the hypothesis that the PAF family of lipid messengers might be associated with cognitive deficits, with strong associations with executive dysfunction driving many of the observed relationships. This study also suggests that the relationship between PAFs and cognitive deficits is strongest in patients with CAD with major depression, implicating PAFs as potentially highly relevant to cognitive decline in that subgroup.

Deficits in executive function are predictive of accelerated cognitive decline and are often observed in depressed individuals [31,32]. For example, executive dysfunction is a hallmark feature of depression associated with vascular risk factors in later life [33]. As such, executive dysfunction may be relevant to the increased risk of cognitive decline in those with CAD and, in particular, patients with CAD with depression. In addition to the risk of cognitive decline associated with executive dysfunction in later life, deficits in that domain are associated with poor health outcomes, such as lower quality of life [34], more frequent falls [35], reduced adherence to medications or general health behaviors [36], and in those with depression, reduced effectiveness of antidepressant interventions [37], among many others. Thus, PAFs may be associated with deficits in this clinically important cognitive domain. Our results extend the previous literature, as several of the PAFs found to be associated with cognitive deficits in this study have established mechanistic relationships with neurodegenerative processes. For example, previous pre-clinical work has identified the PAFs PC(*O-*18:0/2:0) and PC(*O-*16:0/2:0) as important pathological species associated with pro-apoptotic effects in the central nervous system [18]. Furthermore, PC(*O-*16:0/2:0) and its *lyso*-PAF precursor, PC(*O-*16:0/0:0), were found to be elevated in the temporal cortex of patients with Alzheimer’s disease post-mortem [20], collectively implicating the PC(*O-*16:0/2:0) pathway as a potential marker of neurodegeneration.

These results are also consistent with previous literature demonstrating that different PAF species have unique signaling effects both at the PAF receptor (a major conduit for PAF signaling) and independently of the PAF receptor [18]. For example, activation of the PAF receptor by PC(*O-*16:0/2:0) has been shown to be anti-apoptotic, whereas activation by PC(*O-*18:0/2:0) is pro-apoptotic. Additionally, both PC(*O-*16:0/2:0) and PC(*O-*18:0/2:0) have been shown to be pro-apoptotic through different PAF receptor-independent pathways [18]. Thus, each PAF identified in the present study may have a unique signaling potential not only within the periphery, from where it was sampled, but also in the central nervous system, and its effects may be variable depending on whether or not it is activating the PAF receptor. This could explain why the PAF PC(*O-*19:5/2:0) was associated with better global cognitive performance in patients with CAD with depression in this study. For example, it is possible that PC(*O-*19:5/2:0) may signal predominantly at the PAF receptor (or predominantly independently of the PAF receptor) and that its signaling effect may be anti-apoptotic. It is also possible that PC(*O-*19:5/2:0) might be an anti-inflammatory member of the PAF family whose role is to counteract the pro-inflammatory effects of the other PAFs. While caution should be exercised when interpreting the differential associations between PC(*O*-19:5/2:0) and global cognitive performance in patients with and without depression, this PAF may be of mechanistic interest for future study. Unfortunately, the mechanisms by which most of the PAFs identified by this study signal neurodegenerative processes or might be involved in them have yet to be determined. However, the variable negative, neutral, and positive associations between PAFs and cognitive performance might suggest roles for specific PAFs in mechanisms of cognitive decline and it may be possible, through lipidomics, to identify important pathways for future investigation.

These preliminary findings are consistent with previous studies demonstrating an interaction between inflammatory messengers and cognitive deficits in populations without dementia. For example, poorer global cognitive performance has previously been associated with greater concentrations of inflammatory cytokines and acute-phase proteins [3]. Inflammatory markers, such as interleukin-6 and C-reactive protein, have also been associated with poorer performance on tests of psychomotor speed and verbal memory in individuals with depression [38-40]. As such, the role of those markers in the relationship between PAFs and cognitive performance in patients with CAD with depression should be addressed in future studies.

This hypothesis-generating study is novel in several aspects: firstly, these findings are the first indication that PAF metabolism might be linked to cognitive deficits in CAD patients, with high relevance to patients with CAD with depression, a group that is at an elevated risk of accelerated cognitive decline [6]. Secondly, most of the available literature on PAFs does not describe PAF species individually, instead classifying PAFs as a type of lipid (that is, a PAF). Only recently has technology permitted a lipidomic approach to identifying unique PAF species [26,41]. These findings are the first to characterize individual PAF species in plasma from a clinical sample, adding much needed context to their potentially unique roles as bioactive lipid messengers. Finally, the potential of these preliminary findings is enhanced by the lack of confounding associations between participant characteristics and cognitive outcomes.

### Limitations

The interpretation of these findings was limited by the small sample size and cross-sectional study design. Moreover, since the study was intended to generate future hypotheses, no adjustment was made for multiple comparisons. As such, it is possible that the associations (or lack thereof) between certain PAFs and cognitive measures might change in a larger sample, revealing the true associations. As the study population consisted only of older patients with CAD, we cannot comment on the potential associations between PAFs and cognitive deficits in those who are medically healthy, those with depression who do not have CAD, or those younger than 45. This study also did not address measures of other cognitive domains (for example, episodic memory) and therefore these findings cannot be generalized across all cognitive domains. Finally, these findings are limited to PAFs derived from alkylacylglycerophosphocholines; however, as mentioned, these are highly abundant PAFs and are therefore appropriate for this exploratory pilot study.

### Future directions

The PAFs in peripheral blood may indicate a systemic state of aberrant lipid metabolism, owing to pathophysiological processes of cognitive decline or may signal across the blood–brain barrier to initiate synthesis of PAFs and other inflammatory mediators in the central nervous system. If our findings are confirmed in a larger sample, PC(*O-*18:0/2:0) and PC(*O-*16:0/2:0) might be particularly important PAFs to explore in patients with CAD with depression, considering their consistency of involvement with global and domain-specific cognitive deficits and their established mechanistic relevance to neurodegeneration [18,20]. PC(*O-*18:6/2:0) and PC(*O-*19:5/2:0) are newly identified species that represent potential mediators associated with depression-related cognitive deficits in CAD. Should PAFs be confirmed as consistently associated with cognitive deficits, they might serve as clinically useful biomarkers of vulnerability to cognitive decline in patients with CAD. Future studies might clarify the mechanisms by which these PAFs, and others, might mediate neurodegeneration and cognitive decline in patients with CAD, to identify mechanistically relevant therapeutic targets.

In the meantime, these preliminary findings support a role for PAFs as markers of cognitive deficits in CAD patients and invite investigation of this association in larger studies.

## Conclusions

While preliminary, the findings of this study encourage further investigation of PAF species, particularly PC(*O*-18:0/2:0), as markers of cognitive deficits in CAD patients. As PAFs are emerging as important lipid mediators at the core of neurodegenerative pathology, are elevated in CAD, and are associated with leading mechanisms of cognitive decline associated with depression, these findings are biologically plausible and potentially clinically relevant for patients with CAD with depression. Larger longitudinal studies of PAFs as markers of cognitive decline in CAD patients with and without depression might clarify their role and clinical utility as biomarkers or therapeutic targets.

## Abbreviations

ANOVA: analysis of variance; CAD: coronary artery disease; CTPNL: Canadian Institutes of Health Research Training Program in Neurodegenerative Lipidomics; PAF: Platelet-activating factor.

## Competing interests

The authors declare that they have no competing interests.

## Authors’ contributions

GM conceptualized the study, carried out collection, analysis, and interpretation of the data, and drafted the manuscript. NH, SALB, and KLL conceptualized the study, interpreted the data, and revised the manuscript for intellectual content. HX carried out data analysis and revised the manuscript for intellectual content. DF interpreted the data and revised the manuscript for intellectual content. PIO conceptualized the study and revised the manuscript for intellectual content. All authors gave final approval of the version to be published and agree to be accountable for all aspects of the work.
